# A new freshwater snail genus (Hydrobiidae, Gastropoda) from Montenegro, with a discussion on gastropod diversity and endemism in Skadar Lake

**DOI:** 10.3897/zookeys.281.4409

**Published:** 2013-03-28

**Authors:** Vladimir Pešić, Peter Glöer

**Affiliations:** 1 Department of Biology, Faculty of Sciences, University of Montenegro, Cetinjski put b.b., 81000 Podgorica, Montenegro; 2 Biodiversity Research Laboratory, Schulstraße 3, D-25491 Hetlingen, Germany

**Keywords:** Skadar Lake, gastropod endemism, taxonomy, ancient lake

## Abstract

*Karucia sublacustrina* a new species of freshwater snails (Hydrobiidae, Gastropoda) is described based on material collected from Skadar Lake (Montenegro, Albania). The new species belongs to monotypic genus *Karucia* **gen. n.** The shell morphology and body shape of the new genus resembles *Radomaniola* Szarowska, 2006 and *Grossuana* Radoman, 1973, from which it differs in the larger shells with relatively slim and a slightly, but clearly shouldered body whorl. The number of gastropods from Skadar Lake basin tallies now 50 species. The adjusted rate of gastropod endemicity for Skadar Lake basin is estimated to be 38%. By compiling faunal and taxonomic data we also aim to provide information of relevance as to conservation efforts.

## Introduction

The Skadar Lake system is a well-known hotspot of freshwater biodiversity (Pešić et al. 2009) and harbors a highly diverse mollusc fauna (Glöer and Pešić 2008a). Research of gastropod biodiversity on the Skadar Lake has a relatively long tradition since the first records were published by Küster (1843). The history of research of the Skadar Lake gastropod fauna was reviewed by Glöer and Pešić (2008a). As in many of the Balkan lakes, the endemic gastropod species of Skadar Lake were not described until some decades ago. The last recent account of freshwater gastropods of Skadar Lake gave 40 species by number (Glöer and Pešić 2008a). However, modern phylogenetic evaluations are still scarce (e.g. Albrecht et al. 2007, Falniowski et al. 2012) and the lack of such studies hampers discussions on the origin and biogeographical relationships of Skadar Lake mollusc fauna.

During a recent survey of gastropod fauna of Skadar Lake one new hydrobiid genus was discovered and described in the present paper. Therewith, we aim to provide faunal information on Skadar Lake system gastropod diversity and endemism with relevance to conservation efforts.

## The study area

Skadar Lake is the largest lake in the Balkan Peninsula with a surface area that seasonally fluctuates between 370 to 600 km^2^. Skadar Lake itself is located on the western Balkan with approximately two-third (229 km^2^) of its surface belonging to Montenegro and about one-third (142 km^2^) to Albania. The lake’s water level also varies seasonally from 4.7 to 9.8 m above sea level. The lake extends in the NW-SE direction, and it is approximately 44 km long. The Bojana River connects the lake with the Adriatic Sea, and the Drim River provides a link with the Ohrid Lake. The largest inflow is from the Morača, which provides about 62% of the lake’s water. A characteristic feature of Lake Skadar’s water balance is the high inflow from a number of temporary and permanent karstic springs, some of which are sublacustrine in cryptodepressians (so called ‘oko’). The Southern and southwestern sides of the lake are rocky, barren and steep, having bays in which the sublacustrine springs, are usually to be found. On the northern side there is an enormous inundated area, the boundaries of which change as water levels fluctuate.

**Table 1.** Summarized geographical, physiographical, and hydrological characteristics of Skadar Lake (data from Lasca et al. 1981).

Location	42˚03'–42˚21'N, 19˚03'–19˚30'E
Surface area min-max (mean), km^2^	370-530 (472)
Altitude (mean), m a.s.l	5
Length (maximum), km	44
Width (maximum), km	14
Depth (maximum), m	8.3
Depth (mean), m	5.01
Volume	1.931.62×10^6^m^3^
Total drainage area, km^2^	5490
Total length of coastline (including islands) L, km	207
Approximate length of lake outflow (Bojana River), km	40
Climate type	Csa (Koeppen)

**Figure 1.**
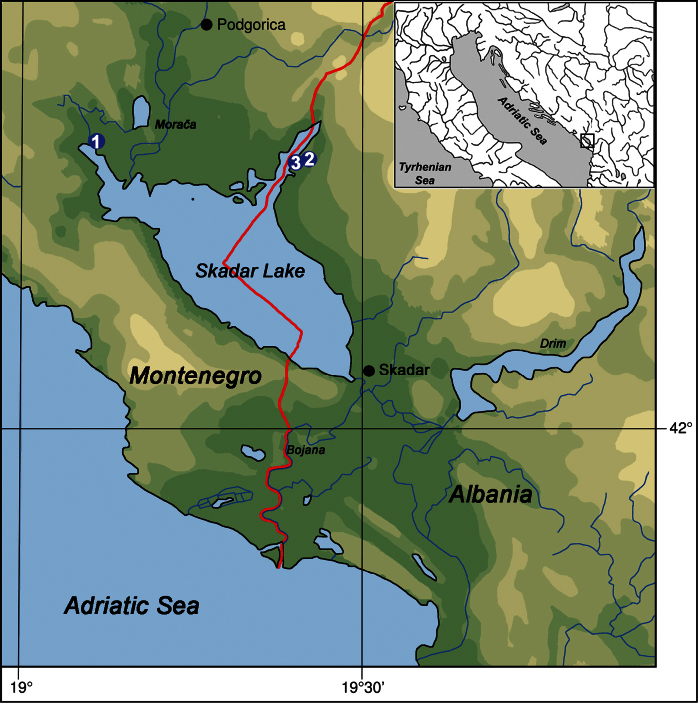
Map of Skadar Lake showing sampling localities of *Karucia sublacustrina* sp. n.: **1** sublacustrine spring Karuč, Montenegro **2** spring Syri i Sheganit, Albania **3** spring Syri i Hurdan, Albania.

## Materials and methods

During field work, gastropods were collected by hand netting, sorted on the spot from the living material andfixed with 80% ethanol. Shell morphometric variables (namely shell height and width) were measured using a stereo microscope (Zeiss). Shells and genital organs were photographed with a Leica digital camera system. The type material is stored in the Zoological Museum of Hamburg (ZMH).

## Results

### Systematics

Family Hydrobiidae Troschel, 1857

#### Genus Karucia

Glöer & Pešić gen. n.

urn:lsid:zoobank.org:act:5A404EE4-A7E9-45A7-80DD-B5737D6047E2

http://species-id.net/wiki/Karucia

##### Description .

Shell large and ovate-conical, with 4.5–5.5 slightly convex whorls. Body whorl relatively slim and prominent, slightly shouldered. The penis is tapered at the distal end and with a bi-lobed outgrowth on the left side.

##### Type species.

*Karucia sublacustrina* sp. n.

##### Etymology.

The genus is named after the type locality.

##### Differential diagnosis.

The new genus appears to be close to *Radomaniola* Szarowska, 2006 and *Grossuana* Radoman, 1973, the hydrobioid genera bearing penis with a bi-lobed outgrowth on the left side and ovate-conical shell with more or less strongly developed last whorl (Radoman 1983). From the aforementioned genera, *Karucia* gen. n. can be distinguished by the larger shells and the characteristic shape of the body whorl which is relatively slim and slightly but clearly shouldered. The shells of the *Radomaniola*/*Grossuana* studied from Greece did not usually exceed 2 mm in height, while some of the shells from Montenegro reached about 3 mm (Falniowski et al. 2012) but significantly below the minimum value established for the specimens of *Karucia* gen. n. Further the shells of the *Radomaniola*/*Grossuana* has tumid body whorl, which is not shouldered.

#### Karucia sublacustrina

Glöer & Pešić sp. n.

urn:lsid:zoobank.org:act:33C8598A-CCB4-46C3-BABA-BDD429513743

http://species-id.net/wiki/Karucia_sublacustrina

Fig. 2a–d, 2k

##### Type series.

Holotype (ZMH 79651): Shell height 3.6 mm, shell width 2.3 mm; MONTENEGRO, Skadar Lake, sublacustrine spring Karuč, 42°21'30.84"N, 19°06'23.03"E, 15.xi.2012 Pešić. Paratypes: 8 ex. ZMH 79652; 20 ex. in coll. Glöer; same data and locality as holotype.

##### Distribution.

Montenegro, Skadar Lake, sublacustrine spring Karuč (Fig. 7a).

##### Further records

(data taken from Zoltán Fehér, Budapest; all material in the collection of the Hungarian Natural History Museum). ALBANIA: Malësi e Madhe district, Bajzë, Syri i Sheganit Spring by the Shkodër (Skadar) Lake, 42°16.360"N, 19°23.757'E, 15 m asl., 17.vi.2012 Fehér, Kovács & Murányi; Malësi e Madhe district, Bajzë, Syri i Hurdan spring lakes near Shkodër (Skadar) Lake, 10 m asl., 42°16.299'N, 19°23.941'E, 17.vi.2012 Fehér, Kovács & Murányi. MONTENEGRO: Cetinje municipality, Karuč, Karuč Spring by the Skadar Lake, 42°21.521'N, 19°06.375'E, 10 m asl., 15.vi.2012 Fehér, Karanović, Kovács, Murányi & Pešić.

##### Etymology.

Named after its occurence in sublacustrine spring.

##### Description.

**Figure 2.**
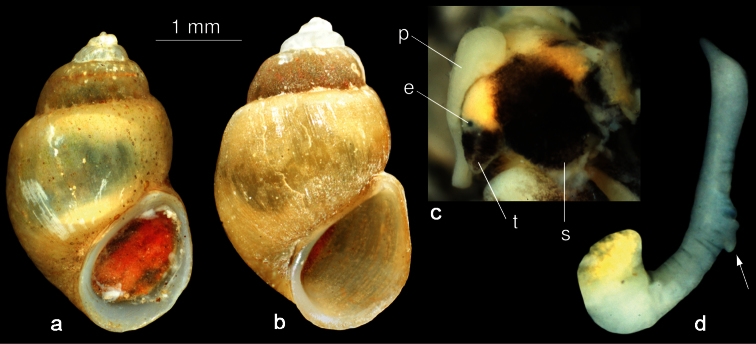
*Karucia sublacustrina* sp. n. **a–b** shells (**a** holotype **b** paratype) **c** head with penis in situ (arrow showing outgrowth on the left side of penis) **d** penis. Abbreviations: **e** eye, **p** penis, **s** snout, **t** tentacle.

The ovate-conical shell consists of 4.5-5.5 slightly convex whorls (Figs. 2a–b). The solid shell is yellowish and silky. The umbilicus is closed. The peristome is sharp and thickened at the columella (Fig. 2a). The aperture is ovoid, somewhat angled at the top. The operculum is orange (Fig. 2a). Body whorl relatively slim but prominent, a little shouldered. Shell height 3.2-4.1 mm, width 2.1-2.6 mm, aperture height 1.7 mm, width 1.4 mm.

The snout and the distal part of the tentacles are dark brown (Fig. 1c). Above the eyes the tentacles at their basis are orange. The penis is tapered at the distal end and bears a bi-lobed outgrowth on the left side (Fig. 2d, arrow).

##### Differentiating features.

As for the genus.

##### Distribution.

Skadar Lake basin (Montenegro and Albania).

### Family Bithyniidae Troschel, 1857

Genus *Bithynia* Leach, 1818

#### Bithynia montenegrina

(Wohlberedt, 1901)

http://species-id.net/wiki/Bithynia_montenegrina

Fig. 3c–d

##### New records.

Montenegro, Skadar Lake area, old stillwater channel near the River Crnojevića and above the village Rijeka Crnojevića, 15 m asl., 42°21.297'N, 19°01.122' E, 10.xi.2012, Pešić.

##### Remarks.

**Figure 3.**
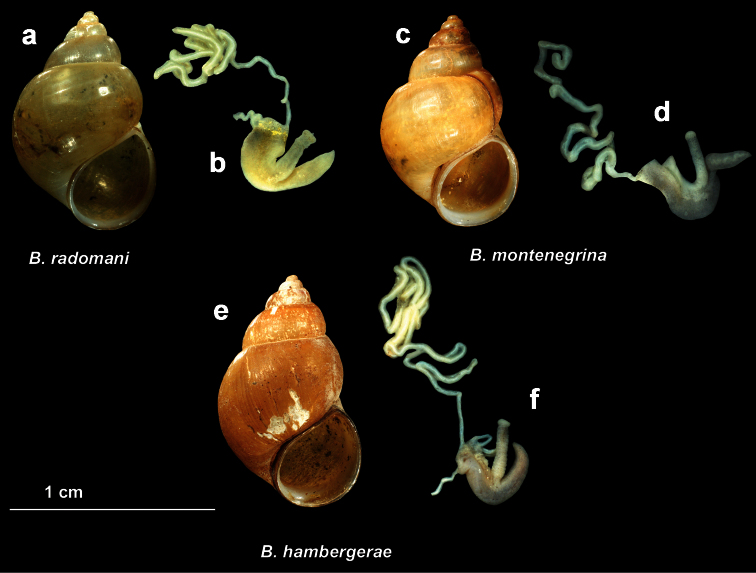
Comparative morphology of shell and penis in *Bithynia radomani* Glöer & Pešić, 2007 (**a–b**) *Bithynia montenegrina* (Wohlberedt, 1901) (**c–d**) and *Bithynia hambergerae* A. Reischütz, N. Reischütz & P.L. Reischütz, 2008 (**e–f**): **a, c, e** = shell, **b, d, f** = penis.

This species was described by Wohlberedt (1901) from River Crnojevića as subspeces of *Bithynia mostarensis*. In the short original description, Wolhberedt (1901) stated that it differs from the nominal form by one additional whorl and the more acute spire. Later on, Reischütz et al. (2008) reported this species from River Crnojevića and mentioned its similarity with *Bithynia radomani* Glöer & Pešić, 2007, a species relatively frequent in the Skadar Lake basin. Recently, we collected *Bithynia montenegrina* in an old stillwater channel near the River Crnojevića, so we were able to examine the morphology of this species and compare it with *Bithynia radomani*. From the latter species, *Bithynia montenegrina* can be distinguished by more slender penis and perennial appendix and the relatively shorter flagellum (compare Fig. 3b and 3d).

**Figure 4.**
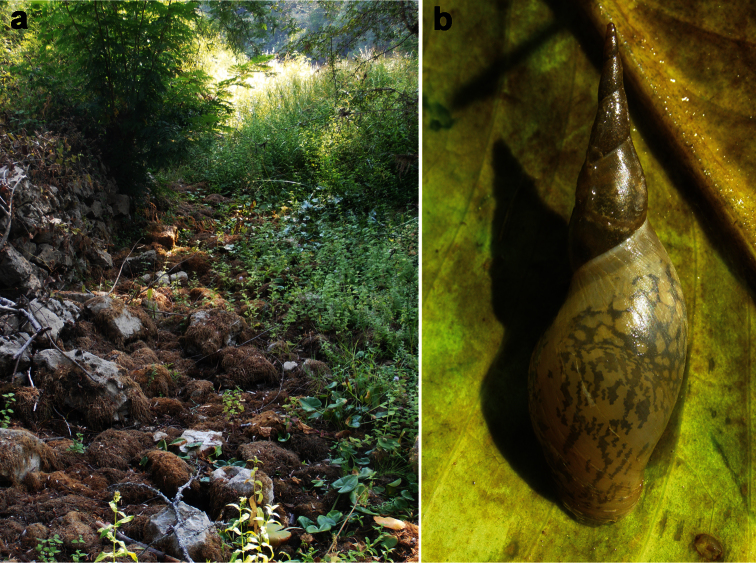
**a** Dry old stillwater channel near the River Crnojevića (September, 2012), sampling site of *Bithynia montenegrina* (Wohlberedt, 1901) **b** *Lymnaea raphidia* (Bourguignat, 1860) from Božaj, Montenegro.

In some females we found specimens with a pseudopenis, a very small, not completely developed penis. This phenomenon is found also in *Bithynia danubialis* Glöer & Georgiev, 2012, a species recently described from the Bulgarian part of the Danube (Glöer and Georgiev, 2012). It is worth to note that most of collected specimens were taken under stones and mud when the old stillwater channel was dry (Fig. 4A).

#### Bithynia hambergerae

A. Reischütz, N. Reischütz & P.L. Reischütz, 2008

http://species-id.net/wiki/Bithynia_hambergerae

Fig. 3e–f

##### New records.

Montenegro, Skadar Lake, Plavnica, River Plavnica, 42°17'03.76"N, 42°12'28.94"N, 15.vi.2012 Fehér, Karanović, Kovács, Murányi & Pešić.

##### Remarks.

*Bithynia hambergerae* was described by Reischütz et al. (2008) from River Plavnica, the northern tributary of Skadar Lake. In the original description, Reischütz et al. (2008) mentioned similarity of this species with the two other *Bithynia* species known from the Skadar Lake basin, i.e., *Bithynia radomani* Glöer & Pešić, 2007 and *Bithynia montenegrina* Wohlberedt, 1901. From the two abovementioned species, *Bithynia hambergerae* differs in the larger dimensions of the shell (12.3–13.8 mm vs. 9.7–11.3 (mean 10.5) mm in *Bithynia radomani*, 10.8–12.6 (mean 11.7) in *Bithynia montenegrina*) and the morphology of penis (moderately slender penis and perennial appendix and the relatively longer flagelum - see Fig. 3f).

### Family Lymaneaidae

Genus *Lymnaea*Lamarck, 1799

#### Lymnaea raphidia

(Bourguignat, 1860)

http://species-id.net/wiki/Lymnaea_raphidia

Fig. 4B

##### New records.

Montenegro: Skadar Lake, sublacustrine spring Karuč, 42°21'30.84"N, 19°06'23.03"E, 10 m asl. Pešić; Skadar Lake, Božaj, pool near spring Vitoja, 42°19'30"N, 19°21'47"E, 8 m asl. Pešić

##### Remarks.

This species was a long time considered as subspecies of *Lymnaea stagnalis* (Linnaeus, 1758). From the latter species, *Lymnaea raphidia* can be easily distinguished by much slimmer spire (Fig. 4b). The preliminary phylogeographic study (Vinarski et al. 2012) shows that populations from Albania and Italy attributed to *Lymnaea raphidia* form a separate clade, distinct from the two other sister clades which corresponds to *Lymnaea stagnalis* and *Lymnaea fragilis*, respectively.

A. and P. Reischütz (2009) mentioned *Lymnaea raphidia* (as *Lymnaea stagnalis raphidia*) from the Montenegrian (Virpazar) and Albanian (Shiroke) part of Skadar Lake.

### Family Ancylidae Rafinesque, 1815

Genus *Ancylus*O.F. Müller, 1773

#### Ancylus recurvus

Martens, 1873

http://species-id.net/wiki/Ancylus_recurvus

Fig. 5b

##### New records.

Montenegro: Skadar Lake, River Gostiljska Reka, 42°17'09.05"N, 19°14'17.35"E, 25.iv.2008 Pešić; River Piva near Mratinje Dam, 43°16'23"N, 18°50'32"E, 20.viii.2010; Pljevlja town, spring in village Vrulja, 21.x. 2010 Pešić; River Zeta near Podgorica, vi. 1982, Glöer.

##### Remarks.

In addition to *Ancylus fluviatilis* we found another *Ancylus* sp. clearly different from the former species by the shell morphology. Already, Pfenninger et al. (2003) and Albrecht et al. (2006a) observed presence of several highly divergent lineages within *Ancylus*. Due to the morphology of the shell our specimens agree well with *Ancylus recurvus* Martens (1873) and detailed description of this species given by Clessin (1882). However, assuming that our studied *Ancylus* belong to *Ancylus* sp. B *sensu* Albrecht et al. (2006) (= Clade 3 of Pfenninger et al. 2003), which geographically cover a wide area (from Canary Islands to Syria), according to Albrecht et al. (2006c) some available older names, such as *Ancylus pileolus* Fèrussac, 1822 would have priority over *Ancylus recurvus*. However, in *Ancylus pileolus* Fèrussac, 1822 the apex is inflated and bent to the left side, while the two other *Ancylus* species mentioned by Albrecht et al. (2006c), i.e., *Ancylus rupicola* Boubée, 1832, and *Ancylus capuloides* “Jan” Porro, 1838, have a small apex which does not reach the border of the shell’s basis, similar as it is depicted in figure of *Ancylus* sp. B by Albrecht et al. (2006b, Fig. 2). Considering Clessin (1882) and Westerlund (1885) the only described *Ancylus* sp. with a straight apex which reaches the border of the shell’s basis as the species from Montenegro, is *Ancylus recurvus*.

**Figure 5.**
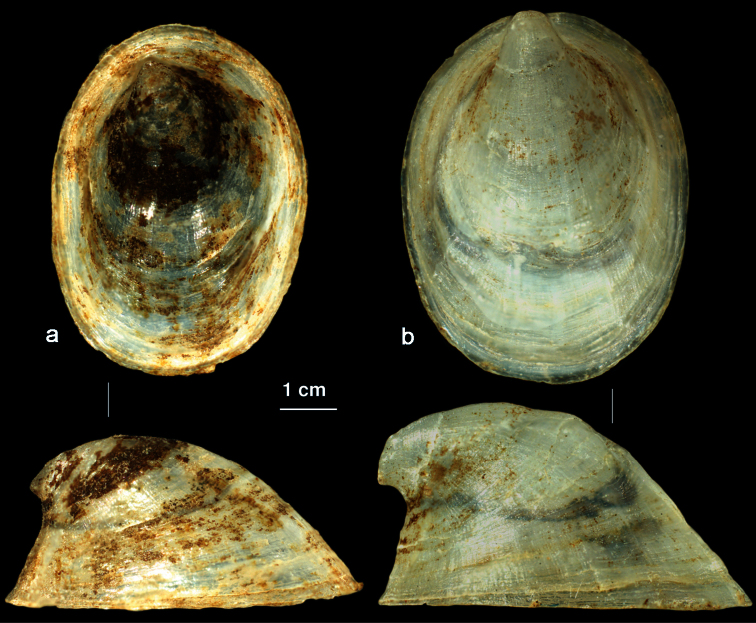
Shell: **a** *Ancylus fluviatilis* (topotype, Germany) **b** *Ancylus recurvus* (Zeta river, Montenegro).

*Ancylus recurvus* can be easily distinguished from *Ancylus fluviatilis* by the shape of apex which is shifted forward reaching the border of the shell’s base (Fig. 5b). Furthermore, the apex in *Ancylus recurvus* is rounded and it is directed more straight, while in *Ancylus fluviatilis* it is acute and turned to the left side.

## Gastropod biodiversity of Skadar lake basin

### Diversity and Endemism

For the Skadar Lake basin, a total of 54 extant gastropod taxa is reported (Glöer and Pešić 2008a, and papers published thereafter, i.e. Glöer and Pešić 2008b, 2009, 2010, Reischütz and Reischütz 2008, 2009, Reischütz et al. 2008) of which we consider at least 50 species to occur in the lake basin (Table 2). In our opinion, four species, i.e., *Stagnicola corvus*, *Gyraulus albus*, *Planorbis planorbis* and *Planorbis carinatus* were incorrectly reported from the Skadar Lake Basin. Former records of *Stagnicola corvus* probably refer to *Stagnicola montenegrinus*, *Gyraulus albus* records refer to *Gyraulus meierbrooki*, while the records of *Planorbis planorbis* and *Planorbis carinatus* refer to *Gyraulus shasi* and *Planorbis vitojensis*, respectively.

At the scale of Skadar Lake, about 31 % of the gastropods (12 out of 39 species sampled in the lake) are endemic. At the scale of the Skadar Lake basin, 38% (19 species) of the total fauna appear to be endemic. Compared with two other famous ancient Balkan lakes (in parentheses % of gastropod endemism, data taken from Albrecht et al. 2009) Ohrid (78%) and Prespa (43%), which gastropod fauna are well studied (Albrecht et al. 2012), Skadar Lake has less taxonomic diversity. Its worth to note, that the number of endemics, however, is likely to change when more faunistical and/or taxonomical data become available. *Stagnicola montenegrinus* orginally was described from Skadar Lake as an endemic species (Glöer and Pešić 2009). However, recently this species was found in the floodplain of the river Maritza in Bulgaria (Schniebs et al. 2012). *Bithynia zeta* is known only from Skadar Lake and one spring near Adriatic coast in Montenegro (Glöer and Pešić 2007), but recently this taxon was found in the Drim River in Albania (P.Glöer, unpublished data).

The on-site molluscan species diversity in the investigated area ranged from one to 14 species, with the highest diversity in the sublacustrine springs. Compared to the other investigated habitats of Skadar Lake lacustrine systems, Karuč was species rich. We found a subset of 14 gastropod species, including eight out of 19 proposed endemic taxa.

Taking lake surface areas into account, Albrecht et al. (2009) gave the index of gastropod endemism of 0.304 (log N_endemic species_/log A_surface area_) for the Skadar Lake. However they take into account 40 species known for the lake and 7 of them being endemic, as well the average maximum surface area (600 km^2^). Based on the revised list of Skadar Lake gastropods and endemics (see Table 2) and the mean surface area (472 km^2^) we get the index of gastropod endemism of 0.478. With this relatively high value, Skadar Lake exceeds such famous lakes as Malawi and Titicaca (see: Rintelen et al. 2007 and, Dejoux and Iltis 1992, respectively).

The faunal relationships of malacofauna among the Balkan’s lakes were analysed by Albrecht et al. (2009). They show that at the species-level, lakes Skadar and Pamvotis (Greece) are clustered as sister group to lakes Trichonis and Lysimachia (both in Greece). Its worth to note that Lake Skadar, inhabited by five *Bithynia* spp. (Glöer and Pešić 2008, Reischütz et al. 2008) is turned out to be a hot spot of *Bithynia* evolution. It’s very likely that the absence of major hydrobioid radiations in some ancient lakes like Skadar, Pamvotis or Trichonis could have triggered diversification in bithyniids (Glöer et al. 2007).

Within the Skadar Lake basin, endemism occurs at different spatial scales: (a) species endemic to Skadar Lake and its sublacustrine springs, adjacent pools as well the mouths of the surrounding tributaries and its downstream parts, (b) species endemic to surrounding springs, (c) species endemic to underground waters (interstitial waters of the surrounding tributaries, and surrounding caves). An estimation of the degree of endemism in the late category show that many endemics are characteristic for the subterranean habitat (Pešić and Glöer 2012).

Skadar Lake endemism occurs also at the genus level. Skadar Lake harbors only endemic and monotypic hydrobiid genus *Karucia* gen. n. Four other Balkan lakes, i.e., Ohrid, Trichonis, Prespa and Mikri Prespa, currently have one endemic genus each (Albrecht et al. 2009).

Despite the still scarce data on the biota of Skadar Lake (e.g. Karanović 2001, Pešić et al. 2010, Pavićević and Pešić 2011, Šundić and Radujković 2012), the currently recognized degree of endemism in different taxa is remarkable, and is not restricted to gastropods but is also evident in some other groups. Talevski et al. (2009) gave total number of 34 native fish species for Skadar Lake and its watershed, with 7 (20.6%) of them being endemic. Karaman (1987) recognized 17 amphipod species for the Skadar lake watershed, 10 of them being endemic (mainly from the subterranean habitat). However, most taxa, remain poorly or even unstudied. Additional field work is highly needed for appropriate evaluation of extant biodiversity of the Skadar Lake.

**Table 2.** Comparative species list and type of endemism of gastropods occurring in Skadar Lake basin. Levels of endemicity: E_skadar_ – endemic to Skadar Lake basin; E_montenegro_ – endemic to the southern and central part of Montenegro; E_montenegro+albania_ - endemic to Adriatic drainage of Montenegro and Albania; E_mont.+alb.+gre._ – endemic to Adriatic drainage of Montenegro, Albania and mainland Greece. Spatial scales of gastropod diversity: LH – species collected in Skadar Lake and its sublacustrine springs, adjacent pools and mouths of the surrounding tributaries (including its downstream part), SH – species collected in the surrounding spring habitat, GH – species living in the subterranean habitat (spr. – found in spring).

	Scale of endemism	LH	SH	GH	Red List Category (after Cuttelod et al. 2011)
Neritomorpha					
*Theodoxus fuviatilis* (Linnaeus, 1758)		+	+		Least Concern
Caenogastropoda					
*Viviparus mamillatus* Küster, 1852	E_mont.+alb.+gre._	+			Data Deficient
*Amphimelania holandrii* (C. Pfeifer, 1828)		+			Least Concern
*Bithynia zeta* Glöer & Pešić, 2007	E_montenegro+albania_	+			Endangered
*Bithynia radomani* Glöer & Pešić, 2007	E_montenegro+albania_	+	+		Least Concern
*Bithynia skadarskii* Glöer & Pešić, 2007	E_skadar_	+			Endangered
*Bithynia montenegrina* (Wohlberedt, 1901)	E_skadar_	+			Data Deficient
*Bithynia hambergerae* Reischütz, N. Reischütz & P.L. Reischütz, 2008	E_skadar_	+			Data Deficient
*Radomaniola curta curta* (Küster, 1852)	E_montenegro+albania_		+		Least concern
*Radomaniola lacustris* (Radoman, 1983)	E_skadar_	+			Critically Endangered
*Radomaniola elongata* (Radoman, 1973)	E_skadar_		+		Critically Endangered
*Radomaniola montana* (Radoman, 1973)	E_montenegro_		+		Least Concern
*Vinodolia scutarica* (Radoman, 1973)	E_skadar_	+			Endangered
*Vinodolia gluhodolica* (Radoman, 1973)	E_skadar_			+(spr.)	Endangered
*Vinodolia matjasici* (Bole, 1961)	E_skadar_			+(spr.)	Critically Endangered
*Vinodolia zetaevalis* (Radoman, 1973)	E_skadar_		+		Data Deficient
*Bracenica spiridoni* Radoman, 1973	E_skadar_			+(spr.)	Endangered
*Karucia sublacustrina* sp. n.	E_skadar_	+			
*Antibaria notata* (Frauenfeld, 1865)	E_montenegro_		+		Least Concern
*Litthabitella chilodia* (Westerlund 1886)			+		Least Concern
*Plagigeyeria montenigrina* Bole, 1961	E_skadar_			+	Critically Endangered
*Plagigeyeriazetaprotogona**** ****vitoja*Reischütz & Reischütz, 2008	E_skadar_			+(spr.)	Endangered
*Pyrgula annulata* (Linnaeus, 1767)		+			Least Concern
Heterobranchia					
*Valvata cristata* O.F. Müller, 1774		+			Least Concern
*Valvata montenegrina* Glöer & Pešić, 2008	E_skadar_	+			Endangered
*Valvata piscinalis* (O.F. Müller, 1774)		+			Least Concern
*Acroloxus lacustris* (Linnaeus, 1758)		+			Least Concern
*Galba truncatula* (O.F. Müller, 1774)		+			Least Concern
*Stagnicola montenegrinus* Glöer & Pešić, 2009		+			Near Threatened
*Radix auricularia* (Linnaeus, 1758)		+			Least Concern
*Radix labiata* (Rossmässler, 1835)		+			Least Concern
*Radix balthica* (Linnaeus, 1758)		+			Least Concern
*Radix skutaris* Glöer & Pešić, 2007	E_skadar_	+	+		Endangered
*Lymnaea raphidia* (Bourguignat, 1860)	E_montenegro+albania_	+			
*Lymnaea stagnalis* (Linnaeus, 1758)		+			Least Concern
*Haitia acuta* (Draparnaud, 1805)		+			Least Concern
*Bathyomphalus contortus* (Linnaeus, 1758)		+			Least Concern
*Planorbarius corneus* (Linnaeus, 1758)		+			Least Concern
*Planorbis vitojensis* Glöer & Pešić, 2010	E_skadar_	+			
*Gyraulus crista* (Linnaeus, 1758)		+			Least Concern
*Gyraulus ioanis* Glöer & Pešić, 2007	E_skadar_	+			Critically Endangered
*Gyraulus meierbrooki* Glöer & Pešić, 2007	E_skadar_	+			Endangered
*Gyraulus shasi* Glöer & Pešić, 2007	E_skadar_	+			Critically Endangered
*Gyraulus* cf. *piscinarum* (Bourguignat, 1852)		+			Not Aplicable
*Anisus vortex* (Linnaeus, 1758)		+			Least Concern
*Hippeutis complanatus* (Linnaeus, 1758)		+			Least Concern
*Segmentina nitida* (O.F. Müller, 1774)		+			Least Concern
*Ferrissia fragilis* (Tryon, 1863)		+			
*Ancylus fuviatilis* (O.F. Müller, 1774)		+	+		Least Concern
*Ancylus recurvus* Martens, 1873		+	+		

### Limnological history of Skadar lake and Gastropod Endemism

**Figure 6.**
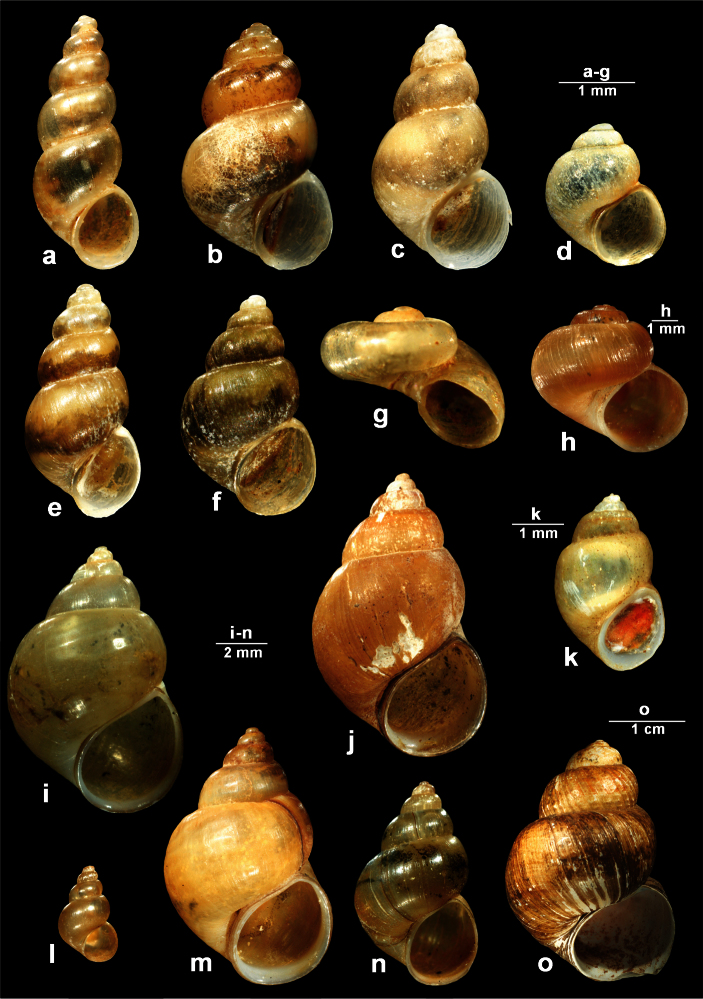
Endemic gastropod species occurring in the Skadar Lake basin – I part. a *Vinodolia matjasici* (Bole, 1961) **b** *Radomaniola curta curta* (Küster, 1852) **c** *Vinodolia scutarica* (Radoman, 1973) **d** *Radomaniola montana* (Radoman, 1973) **e** *Radomaniola elongata* (Radoman, 1973) **f** *Radomaniola lacustris* (Radoman, 1983) **g** *Bracenica spiridoni* Radoman, 1973 **h** *Valvata montenegrina* Glöer & Pešić, 2008 **i** *Bithynia radomani* Glöer & Pešić, 2007 **j** *Bithynia hambergerae* A. Reischütz, N. Reischütz & P.L. Reischütz, 2008 **k** *Karucia sublacustrina* n. gen. n. sp. **l** *Bithynia zeta* Glöer & Pešić, 2007 **m** *Bithynia montenegrina* (Wohlberedt, 1901) **n** *Bithynia skadarskii* Glöer & Pešić, 2007 **o** *Viviparus mamillatus* Küster, 1852.

Most authors agree that the Skadar Lake basin is of tectonic origin (e.g., Laska et al. 1981, Radulović 1997) which had been formed due to the complex folding and faulting within north eastern wing of Old Montenegro anticlynorium (High Karst Zone). These movements took place during the Cenozoic period. The Lake basin has been formed as the result of sinking of blocks in the Neogene period or even in Paleogen. In the Miocene and the Pliocene marine conditions prevailed in the Zeta Plain, which was sunk at the beginning of the upper Miocene, and that the sea inundated this plain up to Podgorica during the Pliocene. Radoman (1985) pointed out that sea must have destroyed all the freshwater populations on this plane and in Skadar Lake area. The connection of Skadar Lake with the sea was interrupted during the younger Pliocene (Radulović 1997). The question of the origin of its water is of particular interest for biologists as these waters may have provided its first species and been the basis for its present high degree of endemism (Albrecht and Wilke 2008). Two hypotheses for the limnological origin of extant Lake Skadar can be advance. In the first scenario Skadar Lake have formed “de novo” in a dry plane (polje) from springs (or rivers) on the place of a former marine gulf. As Radoman (1985) pointed in this scenario, Skadar Lake is a relatively new creation as the whole Zeta plain was a marine gulf until recently which dried up by regression and the lake formed in a dry depression. In the second hypothesis proposed by Radoman (1985), Skadar Lake is probably the remnant of previous, much broader interlinked lacustrine system, first brackish and then freshwater but which were gradually fragmented and disappeared. These remnants are the present day springs and rivers in the sea littoral and in the Skadar depression, as well as in lacustrine continuity – Lake Skadar itself. Radoman (1985) applied the both scenario to explain recent distribution of “lacustrine forms” *Radomaniola lacustris* and *Vinodolia scutarica* in the lake. In the first scenario according to Radoman (1985) *Radomaniola lacustris* and *Vinodolia scutarica* came into existence after the waters of this area become fresh, or after the formation of Skadar Lake, whose vicinity (springs and river) was already inhabited by populations of this genus or ancestral species. In the second scenario, *Radomaniola lacustris* and *Vinodolia scutarica* could be primarily (not secondarily) lacustrine forms (‘remnants’ of the populations from various lacustrine systems on these area), while the river and springs forms were evolved from the lacustrine forms. Radoman (1985) reject the de novo hypothesis mainly based on presence of biogeographical data of hydrobioid gastropods and what he called the presence of the ‘lacustrine forms’ *Radomaniola lacustris* (Fig. 6f) and *Vinodolia scutarica* (Fig. 6c). However, *Vinodolia scutarica* recently was found in a spring in Albania (Fehér and Erőss 2009). Further we found this species in relatively high abundance on the rocky shore of the sublacustrine spring Karuč. *Radomaniola lacustris* was described from sandy bank parts of the lake (Radoman 1983). During our survey from 2005-2012, we could find this taxon at only one sampled locality (sublacustrine spring Karuč). It is very likely that both species are restricted to sublacustrine habitats as the both species inhabit only the southwestern part of the lake, which harbour bays in which the sublacustrine springs are usually to be found. It is worth to note that *Karucia sublacustrina* sp. n. should be considered as an element of spring (sublacustrine?) fauna, because despite the intensive sampling in the lacustrine habitats around the type locality, we could find this taxon only in the sublacustrine spring.

**Figure 7.**
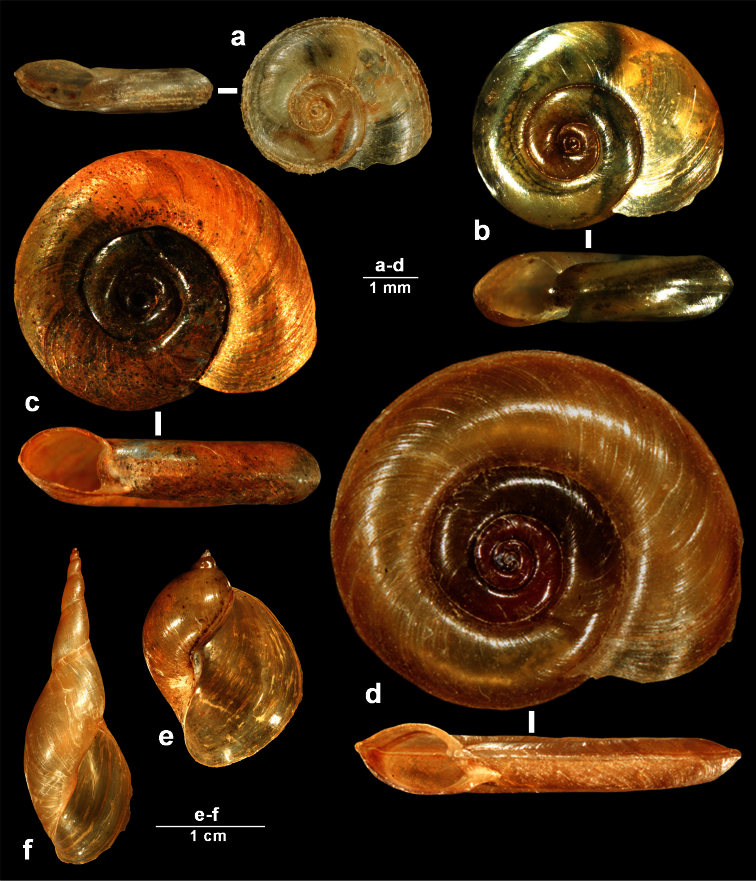
Endemic gastropod species occurring in the Skadar Lake basin – II part. a *Gyraulus meierbrooki* Glöer & Pešić, 2007 **b** *Gyraulus ioanis* Glöer & Pešić, 2007 **c** *Gyraulus shasi* Glöer & Pešić, 2007 **d** *Planorbis vitojensis* Glöer & Pešić, 2010 **e** *Radix skutaris* Glöer & Pešić, 2007 **f** *Lymnaea raphidia* (Bourguignat, 1860).

It should be noted that some other crenobiontic hydrobiid species, inhabitants of neighbouring springs can be also found in lacustrine habitat, at least part of the year as these spring are regularly flooded by the lake water (Fig. 7b). A year study in the spring on the island Vranjina (Fig. 7f), the locus typicus of crenobiontic species *Radomaniola elongata*(Radoman), shows that connection between eucrenon and the lake and consenquently lake water regime is the main factor influnded changes in the benthic assemblages (Šundić and Pešić 2007). The highest number of snails was observed in October during ‘lacustrine phase’ of the spring (the spring was flooded by the lake water), while the snails were absent during the ‘spring phase’ in September when the connection between spring and lake was completely interrupted. This seems to be correlated with development of dump mosses were snails living and the failing water level in the spring (Šundić and Pešić 2007). Falnowski et al. (2012) studied the morphology of the shell, penis, and female reproductive organs, as well as the mitochondrial COI and ribosomal 18S in 17 populations of *Radomaniola* from Skadar Lake drainage and shows that the molecular differentiation was not reflected in morphology. They postulated morphostatic evolution, as a result of non-adaptive radiation characterized by the rapid proliferation of species without morphological and ecological differentiation (Gittenberger 1991).

Nonetheless, information on the potential timing of phylogenetic events on the key endemic taxa in Skadar Lake are still lacking, so the exact limnological origin and the origin of faunal or floral elements of Skadar Lake remain uncertain. Despite the limited knowledge about the lake’s evolutionary history Glöer and Pešić (2008a) presumed status of Skadar Lake as an ancient lake. Although the Skadar Lake is the relatively young ancient lake, his importance for evolutionary research should not be underestimated (Glöer and Pešić 2008). Further studies on gastropods (as well other macrozoobenthic taxa) with applying molecular techniques would certainly give new insights into endemism and evolutionary history of Skadar Lake.

**Figure 8.**
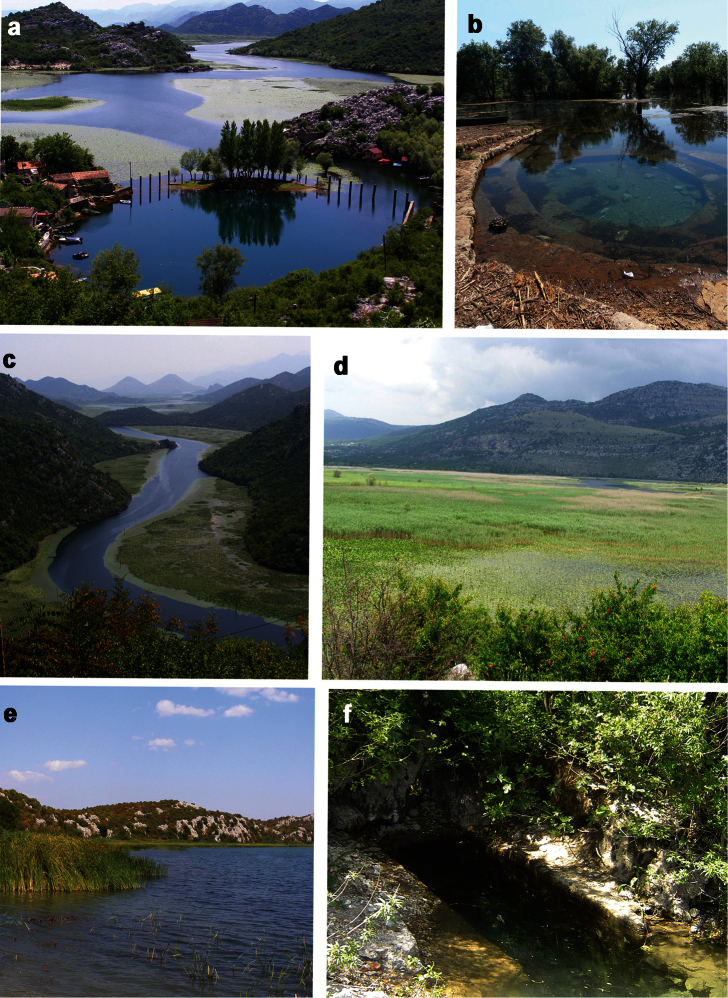
Skadar Lake basin and selected characteristic habitat types. **a** sublacustrine spring Karuč (the locus typicus of *Karucia sublacustrina* sp. n.) **b** spring Vitoja flooded by the lake water (December 2010) **c** View from NE of the lake (River Crnojevića) **d** View from Malo Blato with eutrophic conditions and *Phragmites* belt **e** Šasko Lake (the water of the lake comes from the Bojana River but the communication with the river and the Skadar Lake regulatly interrupred during summer months) – the locus typicus of *Gyraulus shasi* and *Gyraulus ioanis* **f** spring (captured for the local drinking) on the island Vranjina, the locus typicus of *Radomaniola elongata*. Photos. V. Pešić.

## Biodiversity and conservation

Ancient lakes are among the most vulnerable and threatened ecosystems (Lévêque et al. 2005) and these faunas are frequently under extreme anthropogenic pressure (Coulter et al. 2006). The small range of many endemic species living in Skadar Lake system together with ever increasing human pressure make its fauna particularly vulnerable. This becomes even more important in light of ongoing eutrophication, pollution and sand and gravel exploration activities in the lake and its basin. Recently, research of the phytoplankton community and chlorophyllbased trophic state indices (Rakočević-Nedović and Hollert 2005) show that the lake is on a betamesosaprobic level of saprobity, which means moderately polluted with organic compounds.

From a conservation point of view, it is necessary to assess the current status of the endemic species as well to estimate the faunal change during the past decades. However, in most cases this cannot be assessed adequately due to insufficient data so the additional molluscs surveys are necessary even though the species-level taxonomy of many genera are still under discussion (e.g. Falniowski et al. 2012). Regnier et al. (2009) listed *Antibaria notata* as Extinct, presumably based on Falniowski and Szarowska appendix to a paper (Szarowska 2006), which lists the following sites at which they failed to find it in 2001. During our survey, we also could not find this taxon at any of the sampled localities.

Because access to, and sampling in hypogean habitats are difficult, subterranean hydrobiid gastropods have been collected mainly from very few living animals or from empty shells only, and often outside the subterranean networks in springs which flows directly out of the ground (Pešić and Glöer 2012). *Bracenica spiridoni* and *Vinodolia gluhodolica*, the both species presumed to be subterranean forms (Radoman 1983) have been listed as Extinct by Regnier et al. (2009). Zoltán Fehér (pers. comm. 2009) has recorded the latter species in 2000 from one site, 3 km from the original locality, suggesting that the species may be at other sites locally (Pešić 2010). During our survey *Vinodolia gluhodolica* was not found in numerous other springs around the type locality, while the single specimen of *Bracenica spiridoni* were sampled in the sublacustrine spring Karuč.

Effects of human-induced environmental changes are especially evident for sublacustrine springs, with eutrophication and using for water supplying (e.g., sublacustrine spring Karuč) being the most serious threats. Changes are recognizable in the whole ecosystem, for example, by the species loss and invasion by nonnative species. Some endemic species have already gone extinct (e.g., endemic fish *Chondrostoma scodrensis* Elvira, 1987, see Elvira and Almodovar 2008). At the same time, seven nonnative fish species have been introduced into Skadar Lake (Talevski et al. 2009).

These circumstances and the reported decline in endemic gastropod diversity, should trigger efforts to save this sensitive lake ecosystem. The IUCN Red List of Threatened Species (Cuttelod et al. 2011) includes 21 endemic species from the Skadar Lake basin. Six of them are assessed as Critically Endangered, 9 as Endangered, 3 as Data Deficient and 3 as Least Concern in the IUCN Red List of endangered species (see: Table 2). Furthermore, the seven species: *Vinodolia scutarica*, *Vinodolia matjasici*, *Vinodolia zetaevalis*, *Radomaniola lacustris*, *Radomaniola elongata*, *Bracenica spiridoni* and *Valvata montenegrina* are protected in Montenegro by national legislation (Službeni list RCG, br. 76/06, 2006).

## Supplementary Material

XML Treatment for Karucia

XML Treatment for Karucia sublacustrina

XML Treatment for Bithynia montenegrina

XML Treatment for Bithynia hambergerae

XML Treatment for Lymnaea raphidia

XML Treatment for Ancylus recurvus
